# Attachment and Images of Parents and of the Romantic Partner of Russian Young Men and Women

**DOI:** 10.3390/bs10050087

**Published:** 2020-05-08

**Authors:** Natalia Sabelnikova, Dmitry Kashirsky, Olga Garvard

**Affiliations:** 1 Institute of Psychology and Pedagogics, Altai State Pedagogical University, 55 Molodezhnaya str., 656031 Barnaul, Russia; garvard.olga@mail.ru; 2 Department of Criminal Procedure and Criminalistics, Law Institute, Altai State University, 61 Lenin pr., 656049 Barnaul, Russia; psymath@mail.ru

**Keywords:** attachment to parents, attachment to romantic partner, image of a parent, image of a romantic partner, young adulthood

## Abstract

The study investigated young adults’ perceptions of their parents and romantic partners with respect to the quality of attachment to the loved ones. The sample consisted of 78 young Russian men and women aged 19–25 involved in a romantic relationship for at least for 12 months. The employed instruments were the Attachment to Close People Questionnaire (ACOQ), based on the Experiences in Close Relationships (ECR) questionnaire, the Adolescents’ Report of Parental Behavior Inventory, and Leary’s Interpersonal Behavior Measure. Regression analysis was used to analyze the data. The results indicated that the attachment to the mother contributed to attachment to the romantic partner more than the attachment to the father. The attachment security with the partner was associated with the image of the partner.

## 1. Introduction

Establishing deep and healthy relationships with peers, including romantic relationships, is an important developmental task of late adolescence and early adulthood. In addition to self-efficacy, communication, marital relationships, and parenting, close relationships with romantic partners established in late adolescence and young adulthood have a great effect on a person’s social and emotional development at later stages of life [1]. In this regard, choosing a romantic partner as a possible companion in future family life and developing a close relationship with that partner in late adolescence is undoubtedly important.

Attachment theory widely spread abroad and arousing increasing interest among Russian researchers, has often served as an explanatory framework for examining romantic relationships in late adolescence and early adulthood. Attachment theory was originally applied to parent-infant relationships. Bowlby, the founder of the attachment theory, argued that a newborn’s need for close emotional relationships with his parent was caused by environmental threats and served to ensure the child’s biological survival. When getting close to his mother, a child feels comfort and security [2]. The extent of a primary caregiver’s availability and sensitivity shapes a child’s inner working models of attachment, connected with beliefs and expectations about self and others. In cases of high sensitivity and availability of a caregiver, a child develops a secure attachment pattern connected with viewing him/herself as accepted by an attachment figure, as well how available and reliable this attachment figure will be in times of need. Unavailable and unreliable caregiving contributes to a person’s developing insecure attachment patterns. These are also connected to developing a lack of confidence in caregiver support leading one to feel unworthy and unloved. Internal working models start to regulate a child’s attachment behavior towards the caregiver very early (in the first 12 months) and later continue to shape his/her attachment behavior towards other attachment figures, including adults and peers. Ainsworth [3] empirically identified three major attachment orientations in children: secure, anxious–ambivalent, and avoidant attachment. Secure children seek closeness to a caregiver in threatening situations, using them as a secure base. Anxious–ambivalent children preoccupy themselves with a caregiver’s availability. Finally, avoidant children are prone to suppression of their emotions in stressful situations, expressing compulsive self-reliance.

Further research has extended attachment theory to adulthood. Hazan and Shaver’s seminal publication presented an analysis of adult attachment relationships in comparison to early attachments. The authors argued that the emotional bonds that emerged in an adult romantic couple had the same nature and traits as the child’s attachment to his mother. However, there is a difference between child–mother and adult attachments. Both adults involved in a relationship serve as a source of security and comfort for each other in contrast to mother–child relationships, where the mother promotes security for the child, but not vice versa [4]. Recent research literature reviews on the topic [5], including neuropsychological studies, found similarities between romantic couple and infant–caregiver bonds, especially concerning the bond’s features, functions, and dynamics.

Hazan and Shaver [4] empirically identified adult attachment styles similar to those of children, as proposed by Ainsworth. Consequently, researchers [6] conceptualized attachment as a two-factor model. According to this model, attachment styles are a combination of (1) a degree of anxiety concerning close relationships and (2) a degree of avoidance of attachment relationships. According to the model, anxiety is associated with the fear of being unloved by the romantic partner. In turn, avoidance refers to the degree of discomfort with being close to the other. A high degree of avoidance and a low degree of anxiety characterize a dismissive style; a preoccupied attachment is marked by low avoidance and high anxiety. A high degree of both avoidance and anxiety is typical of fearful-avoidant persons; low avoidance and low anxiety is the feature of secure individuals.

A significant number of studies have been devoted to understanding attachment systems’ development through the life-span. Many of these studies stressed the importance of adolescence and young adulthood in the development of close relationships. In adolescence the need for parental dependence declines as adolescents try to establish emotional and behavioral autonomy. Thus, peers and romantic partners gradually begin to meet various attachment related functions [7,8,9]. Meanwhile, research on adolescent and adult attachment has indicated that both broadening attachment functions from parent to peers and attachment to parents play a significant role in adolescent life [9,10,11]. Despite growing autonomous, adolescents and young adults continue to use their parents, especially their mothers, as a source of support in times of stress [2,10,12,13,14]. Attachment security with parents continues to predict a person’s well-being [15]. It has been suggested that young adults have a multidimensional and flexible attachment hierarchy, with either a parent or romantic partner at the top [4].

Theory postulates that replacement of the figures of attachment from parents to peers occurs steadily though late childhood and adolescence [7,11]. Hazan and Zeifman had found that children and adolescents preferred peers over parents for (1) proximity needs and (2) as a source of comfort in times of stress. Only in some cases did transfer of all three attachment functions from parents to peers happen among adolescents [7]. The study reported that 53% of the 6–7-year-old children had transferred the proximity need to peers, about 63% of 11–14-year-old adolescents transferred the safe haven function to peers, and only 40% of 15–17-year-olds preferred their peers as a secure base. Trinke and Bartholomew’s study showed similar results with young adults predominantly choosing peers for safe haven and parents for fulfilling secure base needs [11].

Several researchers [4] argued for the importance of adolescents’ engagement in romantic relationships for transferring of attachment functions from parents to peers. In their longitudinal study of 14–18-year-old adolescents, Friedmeier and Granquist documented that stronger transfer occurred in adolescents engaged in romantic relationships compared with adolescents who had no romantic partners [8]. In addition, Friedmeier and Granquist’s study revealed a link between insecure attachment history and a degree of prospective transfer. Higher degree of transfer was more likely to occur in adolescents who were insecurely attached to their parents. In their adolescent and young adult study, Markiewicz and colleagues [9] found evidence that mothers were preferred to peers as secure bases. However, the researches didn’t find any difference in dynamics of attachment transfer from parents to peers between those who had formed a romantic relationship and those who had not. Similar results were reported by Freeman and Brown [16], who found that mothers continued to be principal attachment figures for secure adolescents aged 16–18 with no relation to having boy/girlfriends as additional attachment figures. Insecure adolescents of the same age demonstrated more preference than secure ones for using a boy/girlfriend as a source of support. The study of Nickerson and Nagle also documented that less secure adolescents were more prone to turn to peers for attachment needs [13]. With respect to adults, several studies [4,11] found the importance of enduring romantic relationships for becoming full-blown attachments. For example, in their study on attachment transfer, Zeifman and Hazan indicated that a romantic couple needs about two years of being together to become a “secure base” for each other [4].

Researchers paid considerable attention to the role which attachment relationships in the family of origin play in later attachment relationships with romantic partners. Mikulincer and Shaver [17] argued that early attachment patterns serve as the platform for the development of subsequent attachment relationships in later life. The empirical research offered support to this idea, showing the contribution of one’s communication with parents at ages 15–16 to his/her security of attachment with romantic partners in adulthood [18]. A number of the recent studies provided inconsistent results concerning the continuity of the quality of attachment through the life-span and the links between attachments to parents and romantic attachments. Some studies documented the influence of the quality of child–mother attachment on the quality of attachment to other people through subjects’ relatively stable inner working models [19,20]. In a similar vein, Furman [21] found similarities between adolescents’ representations of relationships with romantic partners and their representations of relationships with parents and friends. Results of Doyle, Lawford, and Markiewicz’s longitudinal study documented links between insecurity of attachments with mother, best friend, and romantic partner [22]. Doyle and colleagues also found that insecurity of attachment with mother predicted the increase in insecurity with a romantic partner over a two-year period [22]. Dykas and colleagues [23] found the evidence of relation of positive adolescents’ secure base scripts of interaction with parents to secure romantic attachment styles. Along with that, the results of the Dinero and colleagues’ study revealed the connection of the trajectory of romantic relationships in early adulthood with both parent–child relationships and earlier romantic relationships [18]. However, Owens, Crowell, Pan, Treboux, O’Connor, and Waters’ acknowledged only a modest effect of early attachment to parents on conceptualizations of current relationships. The investigators argued that adults’ mental models of their romantic relationships were open to experiences of current attachment relationships [24]. 

A few studies examining specific contributions of each parent to shaping attachment relationships with romantic partners showed no consistent results. According to research [25], relationships of daughters appeared to be closer yet more disputed with mothers than with fathers. Markeiwicz, Lawford, Dolye, and Haggart [9] found that adolescents and young adults are more likely to use mothers than fathers for the secure-base function. Black and Schutte’s study [26] revealed similar effects of relationships with mother and relationships with father on romantic involvement. Several studies documented greater influence of father– rather than mother–child attachment on adolescents’ romantic attachment behavior [27]. In line with these findings, Dalton, Frick-Horbury, and Kitzmann found that among college students, fathers’ parenting was connected with the quality of romantic relationships and mothers’ parenting was not [28]. 

Links between security of attachment to romantic partner and the partner’s psychological characteristics have also received the attention of attachment researchers. Holmes and Johnson [29] reviewed research concerning that topic and came to the conclusion that perceived availability and responsiveness of the partner may be more important for the individuals at the initial stage of relationships, whereas a partner’s perceived love and acceptance towards themselves may be more important at the later stages of relationship development. Holmes and Johnson also found that individuals would ideally like to communicate with secure persons or those who are similar to themselves, in real life; however, they preferred to maintain relationships with partners having a complementary attachment style, who confirmed their expectations of self and others consistently with their internal working models [30].

Less research has been devoted to the development of attachment in transition to adulthood of Russian young adults. The present study is an attempt to provide more information concerning the impact of attachment relationships with parents to selection of attachment partner and to the quality of the romantic attachments among older Russian adolescents and young adults.

The aims of the present study were (1) to compare the associations of attachment to mother/father with the quality of attachment to romantic partner in late adolescence, (2) to examine the investment of one’s perceived image of parents and way of communication with them to his/her security of attachment, and (3) to examine whether parents’ way of communication and the quality of attachment to them predict young adolescents’ selection of a partner and security of attachment to him/her.

## 2. Materials and Methods

The sample consisted of 78 young heterosexual Russian men and women aged 19–25 (*M* = 22.4, *SD* = 1.6). The sample included 64 female and 24 male respondents, who were involved in romantic relationships with the current partner for at least 24 months (*M* = 32, *SD* = 2.3). Only 3 participants were married; 12 were cohabiting; none had children. The data were collected using snowball sampling procedure. All the participants were informed that the participation was anonymous and voluntary, and that they were free to stop answering the survey at any moment if they felt uncomfortable.

The Attachment to Close People Questionnaire (ACOQ, Sabelnikova and Kashirsky [30]), adopted from Experiences in Close Relationships (ECR, Brennan, Clark, Shaver [31]), was used for measuring the quality of attachment to parents and to the romantic partner. The ACOQ scale consisted of 30 items measuring anxiety and avoidance. The Adolescents’ Report of Parental Behavior Inventory (Wasserman, Gorkovaya, Romitsyna [32]) was employed to assess participants’ perception of their parents’ communication with them. It included five scales: positive interest, directivity, autonomy support, hostility, inconsistent strategy of upbringing, difference of positive interest and hostility values, and difference of directivity and autonomy support values. The instruction of the questionnaire was modified to better apply to participants over eighteen years old. Leary’s Interpersonal Behavior Measure, adapted by L.N. Sobchik for the Russian population, was used to gauge respondents’ images of their parents and romantic partners [33]. A demographic information sheet was used for obtaining demographic and personal information (age, gender, length of relationship with the partner, current status of relationships, and satisfaction with relationships).

The obtained data were subjected to correlation and multiple regression analyses. Statistical processing of the results of the study was carried out using the IBM SPSS Statistics 23.0 program.

## 3. Results

### 3.1. Relationships with Parents and Attachment to Romantic Partners

First, we calculated means and standard deviations for avoidance and anxiety on the attachment measure for the parents and for the romantic partner (see Table 1).

Next, we conducted correlation analyses to examine the relationship between attachment to the parents and attachment to the romantic partner on the ACOQ measure. The results of correlation analyses indicated that there was a significant relation between the quality of attachment to romantic partners and the quality of attachment to the mother and to the father.

Avoidance of close relationships with the romantic partner was significantly and positively connected with avoidance of close relationships with the father, r(76) = 0.48; *p* < 0.001, and the mother, r(76) = 0.60, *p* < 0.001. Anxiety concerning the relationships with the mother had positive correlation with anxiety regarding the attachment to the romantic partner, r(76) = 0.79, *p* < 0.001. According to the results of correlation analyses, anxiety in relationships with the father was not connected with the quality of attachment to the romantic partner.

Consequently, we performed a multiple regression analysis (MRA). A backward elimination regression approach was used to identify the independent contribution of attachment to each of the parents to attachment to the partner. The reduced models are presented in Table 2 and Table 3.

The results summarized in Table 2 clearly show that avoidance of close relationships both with the father and with the mother contributes to the model.

The results reported in Table 3 indicate that only anxiety concerning relationships with the mother contributes to the model, but anxiety in regard to relationships with the father does not.

### 3.2. Attachment to Parents and Parent–Target Communication

Means and standard deviations for ADOR scales are shown in Table 4.

We used correlation and multiple regression analyses to examine the connections of attachment to parents with peculiarities of parents’ communication with respondents. Correlation analysis of ADOR scales and the attachment to parents’ domains revealed statistically significant interrelations between respondents’ quality of attachment towards their parents and the perceived parental attitude. Anxiety in relationships with the mother related negatively to the perceived consistency of parental behavioral strategy, r(76) = −0.30; *p* < 0.01, and associated positively with a mother’s autonomy supportive behaviors, r(76) = 0.39, *p* < 0.001. Respondents whose mothers were more inconsistent in their behavior towards their children, and were more autonomy supportive, had more anxiety in their relationship with mothers than those who had less inconsistent mothers. In regard to adolescent–father attachment, the opposite feature was indicated: the more the father supported an adolescent’s autonomy, the less anxiety the adolescent experienced in relationships with him, r(76) = −0.33, *p* < 0.003. Additionally, the positive relation of a father’s criticism to anxious adolescent–father attachment was revealed, r(76) = 0.34, *p* < 0.002. Based on the results of the previous studies [16], we also expected to find the linkage of low-quality communication with parents to the quality of attachment to romantic partners. However, the performed correlation analysis didn’t reveal one.

### 3.3. Attachment to Parents and Romantic Partners and Respondents’ Images of Parents and Romantic Partners

Next, we analyzed the correlations of the quality of attachment to parents with respondents’ images of their parents obtained with Leary’s Interpersonal Behavior Measure. By means of correlation analysis and MRA we revealed the features of the image of the romantic partner and the images of parents which contributed to the quality of attachment to the partner. The findings showed that anxiety concerning the relationship with the mother was connected with a whole range of respondents’ contradictory descriptions of their mother. Thus, anxiety was positively related to descriptions of the mother as dominant, r(76) = 0.38, *p* < 0.001, authoritarian, r(76) = 0.30, *p* < 0.01, and altruistic, r(76) = 0.50, *p* < 0.001. The diversity of inconsistent mothers’ descriptions is in line with the described above data on the inconsistency and low predictability of the mothers of the respondents with anxious-ambivalent attachment to the mother. The avoidance in regard to relationships with the father was connected with the perceived father’s dominance, r(76) = 0.39, *p* < 0.001.

The findings also showed the relationship of the quality of attachment to the parents with the young men and women’s images of romantic partners. The results of correlation analyses for young men and women indicated the association of avoidance of close relationships with the mother and the choice of an authoritarian romantic partner, r(76) = 0.40; *p* < 0.001. The avoidance in relationships with the father related to the choice of an aggressive partner, r(76) = 0.48, *p* < 0.001. Anxiety in the relationships with the father was associated with the choice of an altruistic partner r(76) = 0.37, *p* < 0.001. The image of an authoritarian, r(76) = 0.50, *p* < 0.001, aggressive, r(76) = 0.31, *p* < 0.005, selfish, r(76) = 0.32, *p* < 0.004, and dominant r (76) = 0.35, *p* < 0.002 partner related to the avoidance of close relationships with him/her. Anxiety concerning close relationships with the romantic partner was associated with perceiving the partner as an altruistic person r(76) = 0.37; *p* < 0.001.

Subsequently, we performed a multiple regression analysis using again a backward selection procedure in order to identify the extent of the contribution of each of the particular variables (quality of attachment to the mother and to the father, and the features of the image of the romantic partner to the quality of the attachment to the partner). The full model and the final model coefficients are presented in Table 5, Table 6, Table 7 and Table 8. The results indicate that only the quality of attachment with the mother contributes significantly to the quality of attachment to the romantic partner. 

Table 5 and Table 6 show that avoidance of close relationships with the mother has the most significant contribution to avoidance in relationship with the romantic partner and that the quality of attachment to father does not predict avoidance of close relationships with the partner. The features of the image of the partner do not significantly contribute to avoidance of close relationships with the partner.

The results summarized in Table 7 and Table 8 indicate that anxiety in close relationships with the mother had the most significant contribution to the attachment to romantic partner, comparing with attachment to father and the features of the image of the partner.

## 4. Discussion and Conclusions

The study aimed to examine the investment of attachment to parents, communication with parents, and the perceived parents’ personality features to the choice of attachment partner and to the quality of attachment to the romantic partner in young adulthood. The results indicated that the quality of attachment to each of the parents impacted the quality of attachment to a partner differently. The attachment to the mother contributed to the attachment to the romantic partner more than did the attachment to the father. These results are in line with Bowlby’s hypothesis and some previous empirical research (see for review [34]). Further, the attachment to mother had a greater impact on the attachment to a partner than did the attachment to father. The other features of communication with parents and the features of images of parents and the partner showed a less substantial contribution to the quality of attachment to the romantic partner. The results also indicated that attachment to parents was connected with peculiarities of parents’ communication such as consistency of mother’s behavioral strategy and her autonomy but no impact of the variables related to peculiarities of mother–adolescent communication or father–adolescent communication on attachment to the partner was revealed.

We also found that anxiety and avoidance in close relationships with the partner were significantly associated with a subject’s image of the partner. The authoritarian and selfish features in the image of a partner, perceived aggressiveness and dominance of the partner were connected with avoidance of close relationships with him or her. The results are consistent with the complementarity model of partner preference which postulates that subjects are prone to choose the partners who fit their inner working models of attachment [29,35]. We predicted that mothers and fathers had different influences on romantic partner selection and on the quality of attachment to the partner for young men and for young women but the study didn’t reveal such differences. We suggest that the lack of differences might be due to a small proportion of male respondents in our sample.

Summing up, the findings contribute to understanding the role of the parents in relationships with romantic partners in late adolescence. The obtained results support the findings of other researchers showing that the working models of attachment, formed in childhood, are rather stable and have a tendency to be reproduced in later relationships. The results showed that during late adolescence an individual chooses the partner who fits his working models of attachment to previous attachment figures. The choice of the romantic partner and attachment to him or her also appeared to be attributable to some features of parents’ images and perceived peculiarities of parents’ communication.

Several limitations of this study should be noted. First, the prevalence of females over males in our study may be considered the primary limitation for the precision of conclusions as they might apply more to females than to males. Analyzing differences between males and females needs additional research. Another limitation is the absence of information regarding the history of previous romantic attachment of the respondents. The length and security of their previous romantic relationships may be potential confounding factors as they also may impact the choice of the romantic partner and the security of current romantic relationships. The third limitation is that the study included only individuals who were currently in romantic relationships, possibly excluding people who may be typically avoiding close relationships. Unfortunately, in our study we also couldn’t reveal the contribution of each variable under analysis to the quality of attachment to the partner, taking into account possible mutual connections between the predictors. The sample of the study was not sufficient for testing this hypothesis by means of structural modeling. The next task for further research on the role of attachment to parents, and of the images of the parents and the partner to the quality of attachment to the partner, will be to focus on gender differences.

## Figures and Tables

**Table 1:** Descriptive statistics for attachment security to parents and to the romantic partner *.

ACOQ Scales	Mean	Std. Deviation
Avoidance_Mother	5.42	1.180
Anxiety_Mother	4.75	1.308
Avoidance_Father	4.83	1.238
Anxiety_Father	4.79	1.223
Avoidance_Romantic Partner	5.40	1.126
Anxiety_Partner	4.49	1.274

* the values for avoidance and anxiety ranged from 1 to 7.

**Table 2:** MRA coefficients of the contribution of attachment to each of the parents to avoidance in regard to attachment to the partner *.

Predictors	Unstandardized Coefficients		Standardized Coefficients	t	*p*
	B	Std. Error	β		
Avoidance (Mother)	0.48	0.14	0.50	0.37	0.015
Avoidance (Father)	0.27	0.13	0.29	2.03	0.074

* R^2^ = 0.575, F = 45.67, *p* < 0.0001.

**Table 3:** MRA coefficients of the contribution of attachment to each of the parents to anxiety in attachment relationships with the partner *.

Predictors	Unstandardized Coefficients		Standardized Coefficients	t	*p*
	B	Std. Error	β		
Anxiety (Mother)	0.18	0.16	0.21	1.13	0.001
Anxiety (Father)	0.03	0.17	0.03	1.6	0.372

* R^2^ = 0.678, F = 68.28, *p* < 0.0001.

**Table 4:** Descriptive statistics for communicative characteristics of the mother and the father.

ADOR Scales	Mean	Std. Deviation	ADOR Scales	Mean	Std. Deviation
Positivity (Father)	3.54	1.12	Positivity (Mother)	3.27	1.52
Directivity (Father)	2.51	1.43	Directivity (Mother)	3.03	1.26
Hostility (Father)	2.42	1.00	Hostility (Mother)	2.36	1.41
Autonomy support (Father)	3.78	1.24	Autonomy support (Mother)	3.30	1.18

**Table 5:** Full model coefficients for predicting avoidance of close relationships with the partner *.

Model	Unstandardized Coefficients		Standardized Coefficients	t	*p*
	B	Std. Error	β		
(Constant)	1.47	0.83	-	1.78	0.087
Authoritarian Partner	0.077	0.12	0.24	0.65	0.521
Egoistic Partner	0.010	0.09	0.03	0.11	0.915
Aggressive Partner	0.006	0.11	0.01	0.05	0.958
Dominant Partner	−0.007	0.04	−0.04	−0.19	0.854
Avoidance (Mother)	0.48	0.16	0.50	3.11	0.005
Avoidance (Father)	0.19	0.15	0.20	1.26	0.220

* R^2^ = 0.567, F = 5.464, *p* < 0.0001.

**Table 6:** Final model coefficients for predicting avoidance of close relationships with the partner *.

Model	Unstandardized Coefficients		Standardized Coefficients	t	*p*
	B	Std. Error	β		
(Constant)	2.03	0.69	-	2.93	0.007
Authoritarian Partner	0.09	0.05	0.27	1.90	0.067
Avoidance (Mother)	0.48	0.16	0.50	3.11	0.005

* R^2^ = 0.527, F = 16.149, *p* < 0.0001.

**Table 7:** Full model coefficients for predicting anxiety in regard to close relationships with the partner *.

Model	Unstandardized Coefficients		Standardized Coefficients	t	*p*
	B	Std. Error	β		
(Constant)	0.98	0.59	-	1.69	0.103
Authoritarian Partner	0.01	0.09	0.026	0.10	0.920
Egoistic Partner	−0.11	0.08	−0.269	−1.43	0.161
Aggressive Partner	0.08	0.09	−0.13	−0.83	0.431
Dominant Partner	0.02	0.03	−0.10	0.70	0.488
Anxiety (Mother)	0.82	0.14	0.82	5.89	0.001
Anxiety (Father)	0.03	0.15	0.03	0.19	0.854

* R^2^ = 0.778, F = 14.628, *p* < 0.0001.

**Table 8:** Final model coefficients for predicting anxiety in regard to close relationships with the partner *.

Model	Unstandardized Coefficients		Standardized Coefficients	t	*p*
	B	Std. Error	β		
(Constant)	0.85	0.46	-	1.87	0.001
Egoistic Partner	−0.11	0.04	−0.26	−2.86	0.008
Anxiety (Mother)	0.85	0.09	0.86	9.51	0.001

* R^2^ = 0.763, F = 46.789, *p* < 0.0001.
